# A Potent Antibiotic Combination of Linezolid and Polymycin B Nonapeptide Against *Klebsiella pneumoniae* Infection *In Vitro* and *In Vivo*

**DOI:** 10.3389/fphar.2022.887941

**Published:** 2022-04-26

**Authors:** Ting Huang, Zheng Lv, Jiafu Lin, Kelei Zhao, Longfei Zhai, Xinrong Wang, Yiwen Chu

**Affiliations:** Antibiotics Research and Re-Evaluation Key Laboratory of Sichuan Province, School of Pharmacy, Chengdu University, Chengdu, China

**Keywords:** polymyxin B nonapeptide, linezolid, synergism, *Klebsiella pneumoniae*, antibacterial activity

## Abstract

The emergence of antibiotic resistant Gram-negative bacteria such as *Klebsiella pneumoniae* (KP) is becoming a major public health threat and imposing a financial burden worldwide. A serious lack of new drugs under development is undermining efforts to fight them. In this study, we report a potent combination of linezolid and polymyxin B nonapeptide PBNP (LP) against KP infection *in vitro* and *in vivo*. The checkerboard test and the time-kill assay were performed to detect the antibacterial activity of LP against KP *in vitro*. And the *Caenorhabditis elegans* (*C. elegans*) was used as infection model to evaluate the protective effect of LP against KP infection *in vivo*. The LP combination showed significantly synergistic activity and antibacterial effects against KP, while linezolid and PBNP as monotherapies revealed no dramatically antibacterial activity against the KP strains. Additionally, we found that the LP treatment altered the biofilm production and morphology of KP. Furthermore, the LP treatments significantly protected *C. elegans* from KP infection. In conclusion, this study indicated that the LP combination exhibited significantly synergistic activity against KP and PBNP can be used as a potential activity enhancer. More importantly, this strategy provided the improvement of antibacterial activity spectrum of agents like linezolid and represented a potent alternative to overcome antibiotic resistance in the future.

## Introduction

Gram-negative bacteria, such as *Klebsiella pneumoniae* (KP) is redoubtable threats to public health, leading to huge costs of healthcare worldwide (Li et al., 2016c). KP is a gram-negative, encapsulated, and rod-shaped bacterium and is an extremely important clinical pathogens of hospital infection, causing severe pneumonia, meningitis, and sepsis (Bachman et al., 2015). It is broadly distributed in the environment and mainly settles in human respiratory tract, gastrointestinal tract and urinary tract (Tan et al., 2020). Carbapenem is one of the mainstay of treatment for severe hospital infection caused by KP, while, the drastic increase in the prevalence of carbapenem-resistant KP (CRKP) have caused high morbidity and mortality rates, and leading to a global epidemic in hospitals (Chapelle et al., 2021).

Up to now, the infections with KP or CRKP are resistant to the most antibiotics except polymyxin in worldwide (Doi and van Duin, 2020). Polymyxins were a group of polycationic amphipathic peptide antibiotics and were mainly bactericidal to Gram-negative bacteria by binding its lipid A component of lipopolysaccharide (LPS), permeabilizing the outer membrane, and subsequently inducing the death of cell (Velkov et al., 2013). The polymyxins showed effective activity against a great majority of KP strains, while only two polymyxins (polymyxin B and colistin) are available for clinical application, and are used as the last-line therapy (Li et al., 2006). Moreover, the nephrotoxicity and neurotoxicity of polymyxins are the main concerns preventing their widespread clinical application. Therefore, it is necessary to develop the novel and effective antibacterial agents for the treatment and control of KP infection. To fight the current antibiotic-resistant bacteria, one of the alternative strategies is to combine existing antibiotics with polymyxins by enhancing the efficacy of antibiotics. The ability of polymyxins to exert synergistic effects in combination with lots of antibiotics, including anti-Gram-positive bacterial agents against Gram-negative pathogens or CRKP *in vitro*, has been recently reported (Liu et al., 2014; Di et al., 2015; Wistrand-Yuen et al., 2020). Linezolid is the first member of the synthetic oxazolidinone class antibiotics and has great antimicrobial activity against a wide range of Gram-positive bacteria including staphylococci, streptococci, enterococci (Stalker and Jungbluth, 2003). Linezolid can impede the initiation of bacterial protein synthesis by binding to the 23S RNA peptidyl transferase of the 50S subunit in the bacterial ribosome (Leach et al., 2007). Recently, combined with other antibiotics showed excellent synergistic and antibacterial activity against multidrug-resistant (MDR) pathogens infection (Zhou et al., 2018; Zhuang et al., 2020), suggesting the potential of linezolid as candidate for developing novel combination therapeutic agents. However, combinations of linezolid and polymyxin derivatives for the treatment of KP infections remains an open question.

In the current study, we examined the antibacterial activity of linezolid alone, and in combination with polymyxin B nonapeptide PBNP (LP) against KP *in vitro*. In addition, protective potential of LP was investigated by challenging with KP based on infection model with the murine alveolar macrophages and nematode *Caenorhabditis elegans* (*C. elegans*). The present study provided a novel and effective strategy to control KP infection and facilitated the development of potent therapeutic agents against KP-related diseases.

## Materials and Methods

### Antibacterial Agents

The 11 antibiotics were obtained from the Yuanye (Shanghai, China): including erythromycin, linezolid, lincomycin, Nisin, vancomycin, chlortetracycline, penicillin G, ampicillin, ciprofloxacin, ofloxacin, tigecycline. The polymyxin B heptapeptide (PBHP) was obtained from Nanjing Peptide Biotech (Nanjing, China) and polymyxin B octapeptide (PBOP) was obtained from Dayang Chem (Hangzhou, China). The polymyxin B nonapeptide (PBNP) were purchased from MedChem Express (NJ, United States).

### Bacterial Strains, Cell Lines, *Caenorhabditis elegans* Strain and Growth Conditions

The *Klebsiella pneumoniae* type strain ATCC 13883 (KP13883) was obtained from the American Type Culture Collection and maintained as the manufacturer’s instructions described. The clinical strain carbapenem-resistant *K. pneumonia* 25826 (KP25826) was kindly provided by Dr. Zongxin Fang (The First People’s Hospital of Hefei). The clinical strain hypervirulent *K. pneumonia* (hvKP) NTUH-K2044 (KP 2044) and *K. pneumonia* WT strain ATCC 43816 (KPWT) were kindly provided by Dr. Min Wu (University of North Dakota) and Dr. V. Miller (University of North Carolina) and were cultured in LB (Luria-Bertani) broth/agar at 37°C as described previously (Lawlor et al., 2005; Li et al., 2016a; Li et al., 2016b). The murine alveolar macrophage cell line MH-S cell was preserved in the laboratory and cultured as previously described (Zhao et al., 2019). The *C. elegans* strain N2 was preserved in the laboratory (Li et al., 2021) and cultured at 20°C on the nematode growth medium by feeding with *Escherichia coli* (*E. coli*) OP50 stain as a regular diet.

### Susceptibility Tests

The MICs (minimal inhibitory concentration) were detected for the *K. pneumonia* strains by dilution method, with a final concentration of 1 × 10^5^ CFU/ml on LB medium for 18–24 h, as previously described by the Clinical and Laboratory Standards Institute (CLSI, 2014). All the experiments were carried out in triplicate.

### Antibiotic Synergy Test

The synergy of three polymyxin B derivatives (PBHP, PBOP, PBNP) combined with five antibiotics were tested by the checkerboard method as previously described (Magi et al., 2015). All the experiments were performed in triplicate. Briefly, a two-dimensional array was performed to prepare the serial concentrations of test compounds according to the MIC of the two compounds. The output of checkerboard test was carried out and to obtain the Fractional Inhibitory Concentration Index (FIC) based on the formulas: FIC Index = FIC_A_ + FIC_B,_ FIC_A_ = MIC_A+B_/MIC_A_, FIC_B_ = MIC_B+A_/MIC_B_. The value of MIC_A+B_ is the MIC value of compound A with compound B, and likewise for MIC_B+A_. The FIC Index values were indicated as shown below: synergy (FIC Index ≤0.5), antagonism (FIC Index >4.0), and no interaction (FIC Index >0.5–4.0).

### Biofilm Production

The production of biofilm was detected by crystal violet staining as previously described (Wu et al., 2012). Briefly, 1.0 × 10^6^ CFU of KPWT were cultured in 24-well plate or glass tubes with appropriate LB broth and treated with phosphate-buffered saline (PBS, pH 7.4), linezolid (16 μg/ml), PBNP (8 μg/ml) or combination of linezolid and PBNP (LP) at 37°C for 12 h, respectively. The unattached bacteria were removed and the plates or tubes were washed three times with PBS. The biofilms were stained with 0.2% (wt/vol) crystal violet for 30 min and detected at OD_595_ after dissolution by 95% ethanol. All the experiments were carried out in triplicate.

### Scanning Electron Microscopy

The 1.0 × 10^6^ CFU of KPWT were cultured in 12-well plate with LB broth and treated with PBS, linezolid, PBNP or LP at 37°C for 12 h, respectively as described above. The morphological and surface characteristics were detected by scanning electron microscopy (SEM) as previously described (Rajeshwari et al., 2009).

### Infection Experiments in MH-S Cell Model

The KPWT strain was grown for 14 h in LB broth at 37°C. The KPWT pellet was prepared by centrifugation at 5,000 g and then was resuspended in sterile PBS for infection. The MH-S cells were grown in RPMI-1640 medium containing 10% fetal bovine serum (FBS) at 37°C with 5% CO_2_. The infection experiments in MH-S cells were performed according to a previously report (Ye et al., 2015). Briefly, the MH-S cells were changed to an antibiotic-free medium and infected by KPWT at an MOI of 10:1 (bacteria-cell ratio) for 1 h, and treated with PBS, linezolid, PBNP or LP for 1, 6, and 12 h respectively as described above. The bacterial burdens in the MH-S cells were detected at different time point as previously described (Huang et al., 2020).

### Infection Experiments in *Caenorhabditis elegans* Model

To further investigate the protective efficacy of LP, the *C. elegans* were infected with KPWT or KP25826 (10 worms/group) as previously described (Kamaladevi and Balamurugan, 2017). The infected worms were transferred to the 24-well culture plate containing sterile M9 buffer and then treated with PBS, linezolid (16 μg/ml), PBNP (8 μg/ml) or LP, respectively. The mortality of the challenged *C. elegans* was monitored for the subsequent 7 days. The nematodes that did not show obviously pharyngeal pumping and response to touch were indicated as dead. The bacterial burdens of KPWT or KP25826 in the *C. elegans* were detected at 12 or 24 h post infection. All the experiments were performed in triplicate.

### Statistical Analysis

The Data and statistical tests were analyzed by using GraphPad Prism 6.0. A Mantel-Cox log rank test was applied to compare the survival rates between the control group and monotherapy-treated or LP-treated *C. elegans* (Huang et al., 2019). Means were compared by using a one-way analysis of variance (ANOVA), followed by a Tukey–Kramer post hoc test using a 95% confidence interval.

## Results

### Headings Antibiotic Sensitivity and Resistance Patterns

As shown in Table 1, all the tested KP strains were highly resistant the conventional antibiotics which were used to kill Gram-positive bacteria (including erythromycin, lincomycin, linezolid, nisin, vancomycin). In addition, all the strains were susceptible to the conventional antibiotics against Gram-negative bacteria in a low concentration pattern (penicillin G, ampicillin, ciprofloxacin, ofloxacin, tigecycline, chlortetracycline). However, all the tested strains were highly insusceptible with the 3 different polymyxin B derivatives (PBHP, PBOP, PBNP).

**TABLE 1:** MICs of 4 *K. pneumonia* strains tested by different antimicrobial agents.

Antimicrobial Agents	MIC (μg/ml)			
	KP13883	KPWT	KP2044	KP25826
Erythromycin	>256	>256	>256	>256
Lincomycin	>256	>256	>256	>256
Linezolid	>256	>256	>256	>256
Nisin	>256	>256	>256	>256
Vancomycin	>256	>256	>256	>256
Penicillin G	8	4	16	32
Ampicillin	4	8	8	8
Ciprofloxacin	1	2	4	16
Ofloxacin	2	1	8	16
Tigecycline	4	4	8	8
Chlortetracycline	4	4	16	16
PBHP	256	256	256	256
PBOP	256	256	256	256
PBNP	256	256	256	256

### Strong Antibacterial Synergy of Combined Polymyxin B Derivatives and Antibiotics

In the checkerboard assay, 15 different combinations of three polymyxin B derivatives and five conventional anti-Gram-positive agents were tested for *K. pneumoniae* (Table 2). FIC Index values were calculated by considering all combinations of three polymyxin B derivatives and five conventional antibiotics in which there was no visible growth. The lowest FIC Index value was shown in each combination group as indicated in Table 2, which were ranged from 0.063 to 0.500 in KP plates. The synergy (FIC Index ≤0.5) was detected in all strains and antagonism was not observed. Consideration that combinations of linezolid (16 μg/ml) and PBNP (8 μg/ml) (LP) had the lowest FIC index in all the tested group, we focused on LP as a novel combination and further investigated their antibacterial activity against KP throughout the present study.

**TABLE 2:** FICs of three polymyxin B derivatives combined with five antibiotics against *K. pneumonia*.

AMA	MIC (μg/ml)		FIC	AMA	MIC (μg/ml)		FIC	AMA	MIC (μg/ml)		FIC	AMA	MIC (μg/ml)		FIC	AMA	MIC (μg/ml)		FIC
	SGL	BC^a^			SGL	BC^a^			SGL	BC^a^			SGL	BC^a^			SGL	BC^a^	
PBHP	256	64	0.28	PBHP	256	32	0.18	PBHP	256	16	0.12	PBHP	256	64	0.50	PBHP	256	16	0.12
ERY	512	16		LIN	512	32		LE	512	32		NI	512	128		VAN	512	32	
PBOP	256	32	0.18	PBOP	256	16	0.12	PBOP	256	16	0.09	PBOP	256	64	0.37	PBOP	256	16	0.09
ERY	512	32		LIN	512	32		LE	512	16		NI	512	64		VAN	512	16	
PBNP	256	32	0.25	PBNP	256	32	0.15	PBNP	256	8	0.06	PBNP	256	64	0.31	PBNP	256	32	0.18
ERY	512	64		LIN	512	16		LE	512	16		NI	512	32		VAN	512	32	

a Combination of polymyxin B derivatives and antibiotics giving the lowest FIC, Index value. Abbreviation: AMA, antimicrobial agents; SGL, Single-use; BC, best combination; FIC, fractional inhibitory concentration index; ERY, Erythromycin; LIN, lincomycin; LE, linezolid; NI, nisin; VAN, vancomycin.

### LP is Highly Antibacterial in Culture Media

**FIGURE 1:**
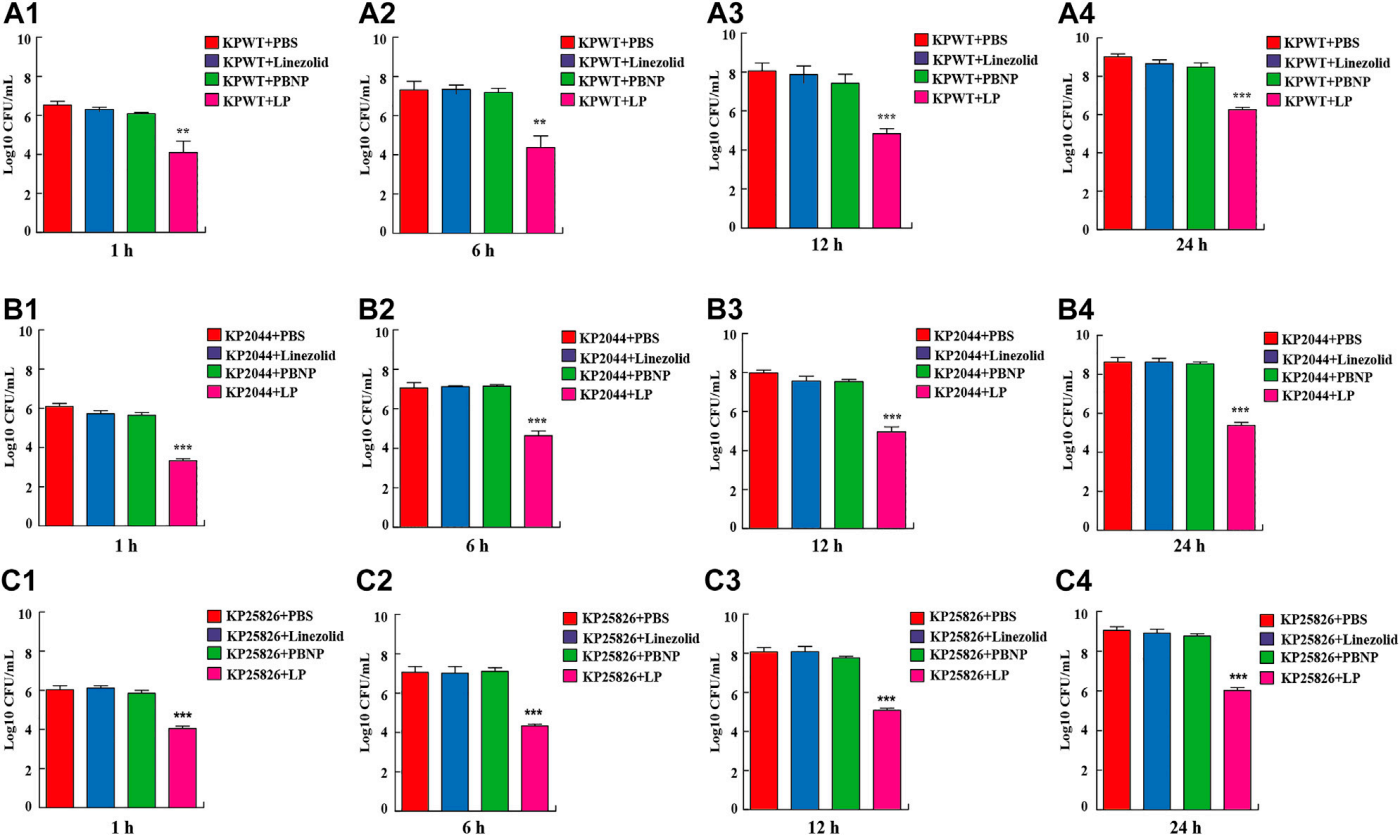
Treatment of LP reduced *K. pneumoniae* viability **(A1–A4)** 1 × 10^5^ CFU of KPWT were seeded in 96-well plates and treated with PBS, linezolid (16 μg/ml), PBNP (8 μg/ml) or combination of linezolid and PBNP (LP) for 1 h **(A1)**, 6 h **(A2)**, 12 h **(A3)**, and 24 h **(A4)**, respectively. The bacteria on the LB agar plates were counted. **(B1–B4)** 1 × 10^5^ CFU of KP2044 were seeded in 96-well plates and treated with PBS, linezolid (16 μg/ml), PBNP (8 μg/ml) or LP for 1 h **(B1)**, 6 h **(B2)**, 12 h **(B3)**, and 24 h **(B4)**. **(C1–C4)** 1 × 10^5^ CFU of KP25826 were seeded in 96-well plates and treated with PBS, linezolid (16 μg/ml), PBNP (8 μg/ml) or LP for 1 h **(C1)**, 6 h **(C2)**, 12 h **(C3)**, and 24 h **(C4)**. The bacteria were determined as described above. Data are shown as the mean ± SEM of three independent experiments. ***p < 0.001, **p < 0.01.

To investigate the antibacterial activity of LP in the *K. pneumoniae in vitro*, KPWT strains were seeded in 96-well plates and treated with PBS, linezolid, PBNP or LP for 1, 6, 12, and 24 h, respectively. The results showed that bacterial CFU was decreased significantly in the LP treated group compared to the PBS-treated group at different time point (Figures 1A1–A4). To validate these data, we further tested the antimicrobial effects of LP on the hypervirulent clinical strain KP 2044 (Figures 1B1–B4) and clinical CRKP strain KP25826 (Figures 1C1–C4). Treatment with LP significantly decreased the CFU of KP at the indicated time points compared the linezolid-treated or PBNP-treated group (Figures 1B1–B4,C1–C4).

### LP Altered Biofilm Production and the Morphology of *Klebsiella pneumoniae*

**FIGURE 2:**
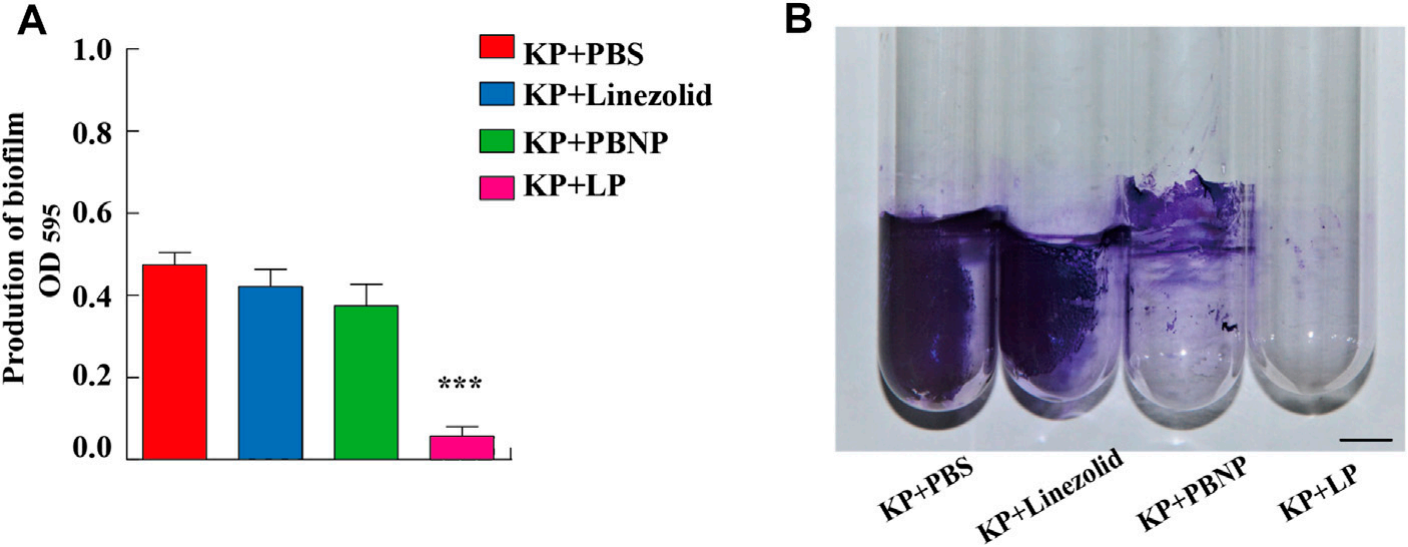
The biofilm production of *K. pneumoniae* treated by LP. **(A,B)** 1 × 10^6^ CFU of KPWT or were seeded in 24-well plates **(A)** or glass tubes **(B)** and treated with PBS, linezolid (16 μg/ml), PBNP (8 μg/ml) or LP for 12 h. The biofilm production was detected at OD_595_ by microplate reader. Scale bar, 1 cm. Data are shown as the mean ± SEM of three independent experiments. ***p < 0.001.

**FIGURE 3:**
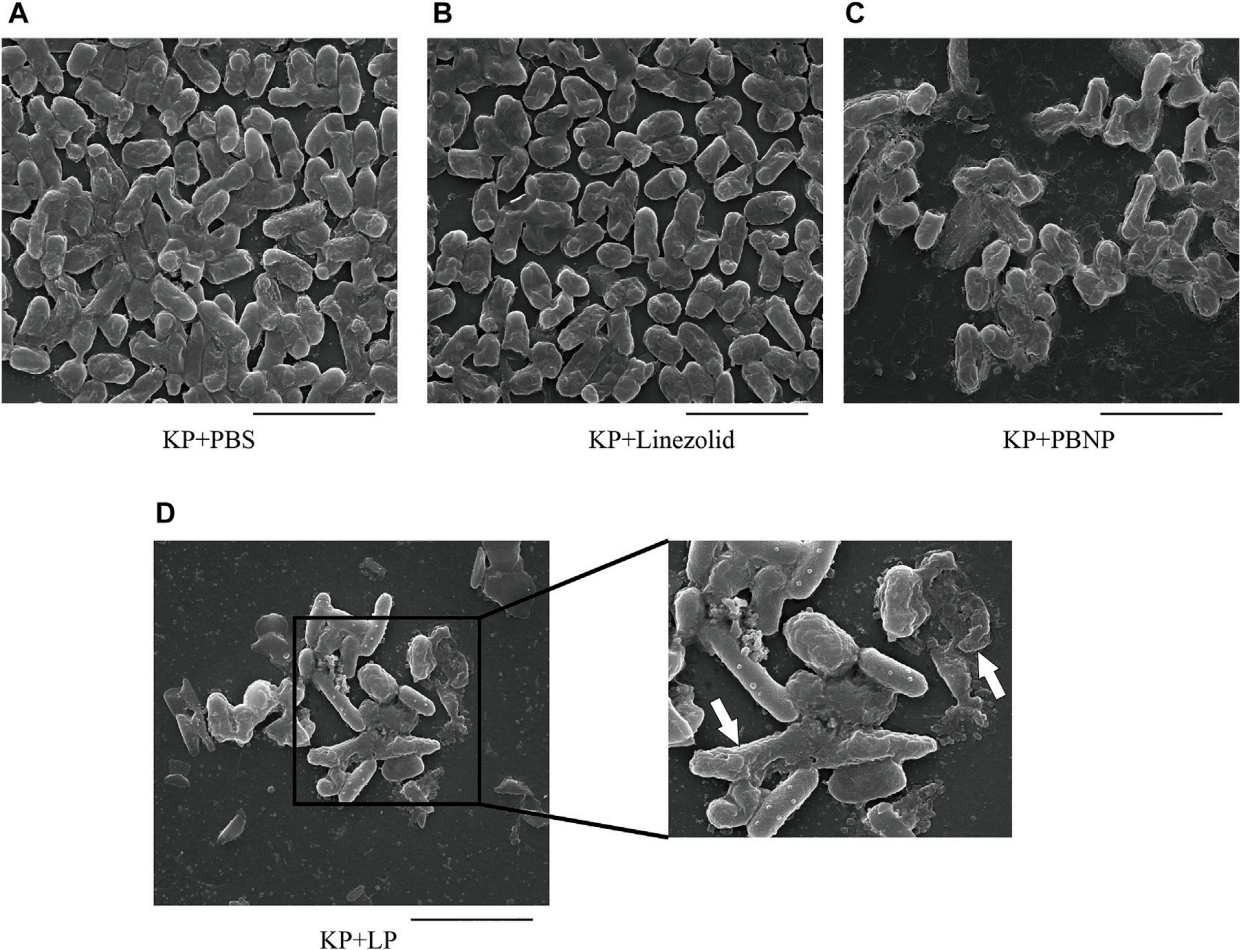
The morphology of *K. pneumoniae* treated by LP **(A–D)** 1 × 10^5^ CFU of KPWT were seeded in 24-well plates and treated with PBS **(A)**, linezolid (16 μg/ml) **(B)**, PBNP(8 μg/ml) **(C)** or LP **(D)** for 12 h. The morphology of KPWT was detected by scanning electron microscopy (SEM). The destroyed KPWT is indicated by the arrow. Scale bar, 5 μm. All the experiments were performed in triplicate.

*K. pneumoniae* predominantly form biofilms, which are extremely hard to eradicate with currently commercial antimicrobial agents. To explore whether LP treatment could alter the biofilm production of *K. pneumoniae*, KPWT were seeded in 24-well plates or glass tubes and treated with PBS, linezolid, PBNP or LP for 12 h. As shown in Figures 2A,B, the LP treatment could significantly reduce the biofilm production of KPWT. Next, to investigate whether LP could affect the morphology of *K. pneumoniae*, KPWT were cultured in 12-well plates and treated with PBS, linezolid, PBNP or LP for 12 h. Our results showed that the LP significantly affected the morphology of KPWT such as cell shrinkage or cell lysis by SEM (Figure 3D), while the morphology of KPWT in the control group or monotherapy-treated groups did not show obviously changed (Figures 3A–C).

### LP Reduced Bacterial Burdens in MH-S Cell Model *In Vitro*

**FIGURE 4:**
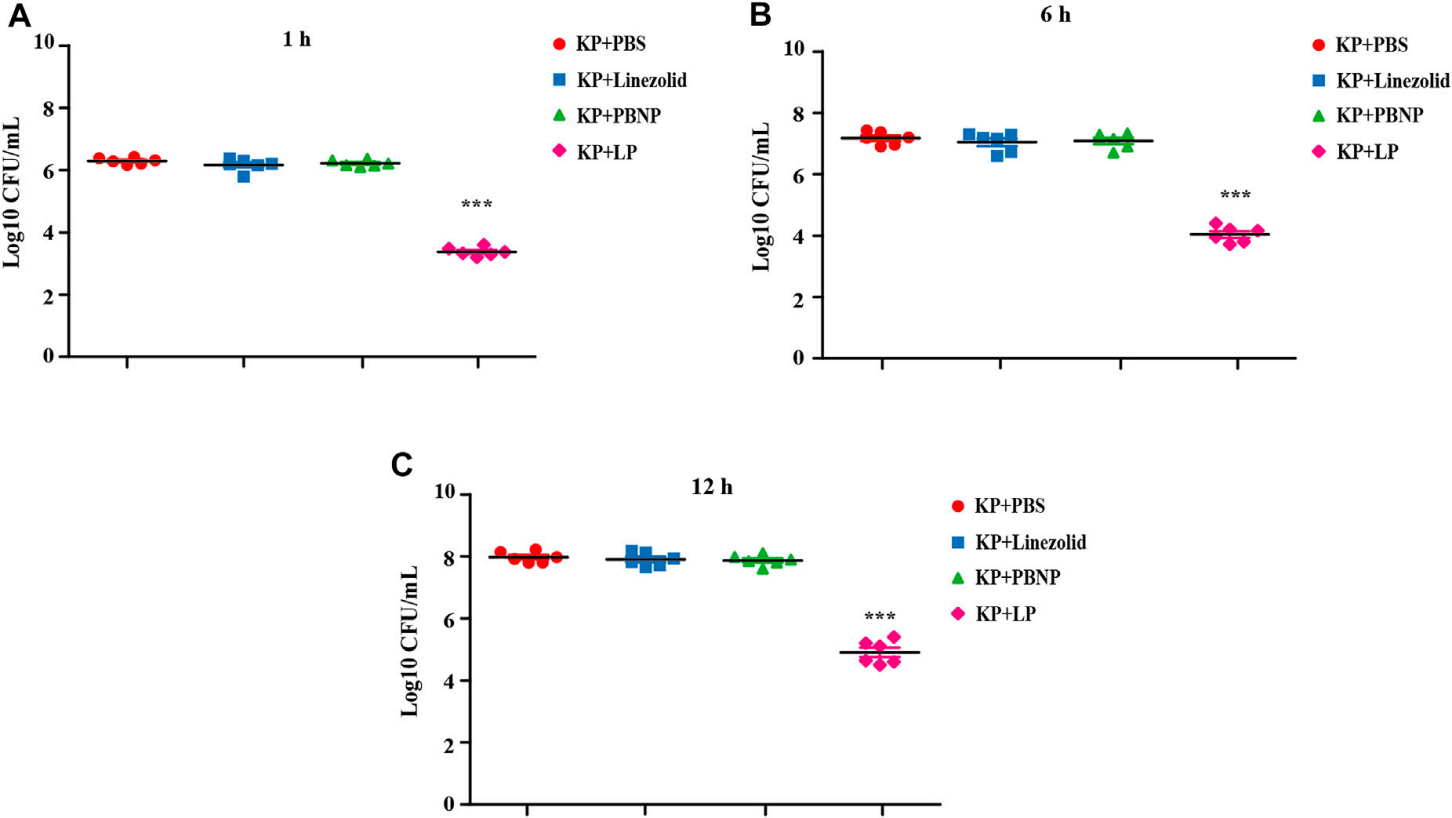
Bacterial burdens in MH-S cell infected with *K. pneumoniae* **(A–C)** The MH-S cells were infected with KPWT at an MOI of 10 for 1 h. And then treated with PBS, linezolid (16 μg/ml), PBNP (8 μg/ml) or LP for 1 h **(A)**, 6 h **(B)**, and 12 h **(C)**. The bacteria burdens were determined as described above. Data are shown as the mean ± SEM of three independent experiments. ***p < 0.001.

As one of the most important cell types in the antibacterial immunity, macrophages are the predominant cells against bacterial infection. To define the collective role of LP against KPWT infection, we next examined the antibacterial effects of LP on KPWT in a MH-S cell model. MH-S cells were infected with KPWT for 1 h, and then treated with PBS, linezolid, PBNP or LP for 1, 6, and 12 h respectively. The results showed that the viability of KPWT in the monotherapy-treated groups were no statistically significant differences compared to the PBS group at indicated time point (Figures 4A–C). However, LP significantly increased elimination of KPWT in MH-S cells from 1 to 12 h post infection, suggesting that LP protected cells from *K. pneumoniae* infection.

### LP Protected *Caenorhabditis elegans* From *Klebsiella pneumoniae* Infection

**FIGURE 5:**
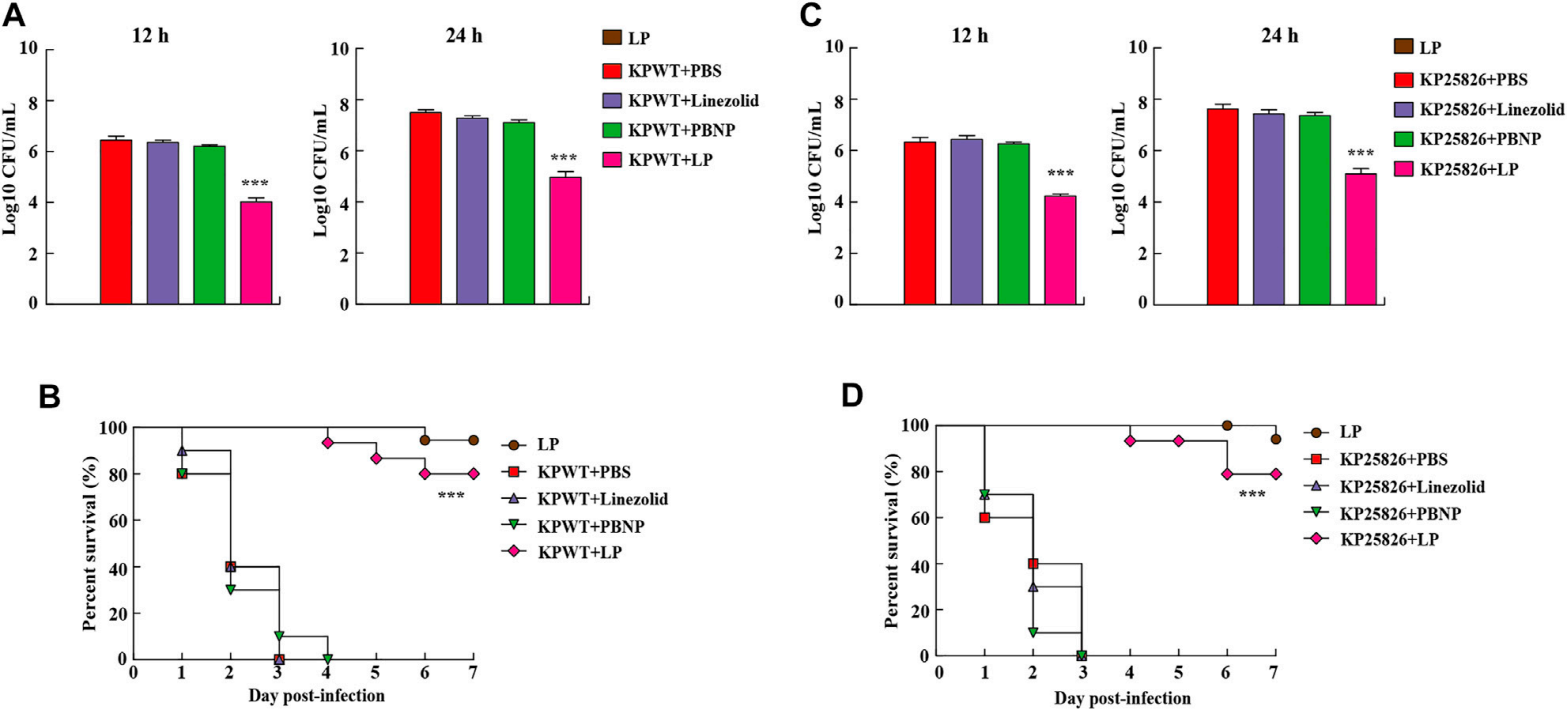
Residual CFUs and survival rate of *C. elegans* infected with *K. pneumoniae*. The *C. elegans* were infected at day 0 with KPWT or KP25826 and treated with PBS, linezolid (16 μg/ml), PBNP (8 μg/ml) or LP for determining bacterial burdens at post treatment 12 and 24 h **(A,C)**. The survival rate of *C. elegans* was monitored for the subsequent 7 days **(B,D)**. PBS was used as a control. ***p < 0.001.

The nematode *C. elegans* is a simple and efficient model host for high-throughput screening of anti-infective agents in KP. To further investigate the antibacterial effect of LP *in vivo*, the *C. elegans* were infected with KPWT or clinical CRKP strain KP25826 and then treated different drugs. We found that large amount of bacteria from the *C. elegans* were recovered in the PBS, linezolid or PBNP-treated groups (Figure 5A). While, the *C. elegans* treated with LP displayed few bacteria inside the *C. elegans* at 12 and 24 h post infection (Figure 5A). As shown in Figure 5B, the PBS, linezolid or PBNP treated-*C. elegans* died at day 3 or day 4. However, most *C. elegans* survived the KP challenge at days 7 post infection by treated with LP. Importantly, most *C. elegans* from LP group without infection survived, suggesting LP has no obvious drug toxicity in *C. elegans*. To further validate the data, we also tested the antimicrobial effects of LP on clinical CRKP strain KP25826 (Figures 5C,D). Treatment with LP significantly decreased the bacterial CFU at the indicated time points compared the control group (Figure 5C) and protected *C. elegans* against *K. pneumoniae* infection (Figure 5D).

## Discussion

The MDR gram negative bacteria are becoming increasingly predominant, for which there is lack of antibacterial agents to fight them in the current antibiotic pipeline (de la Fuente-Núñez et al., 2015). For example, to treat the infections caused by the MDR-KP is difficult because of the adaptive resistance of this pathogen to most commercial antibiotics (Chapelle et al., 2021). Antibiotic combination therapy is currently applied for some severe infections caused by *K. pneumoniae* (Tan et al., 2020; Wistrand-Yuen et al., 2020), while useful data regarding which combinations are most potent are still limited. In the current study, we investigated the synergistic effects of polymyxin B derivatives and 5 other antibiotics against *K. pneumoniae* and reported the significantly positive interactions in combination of linezolid and polymyxin B nonapeptide PBNP (LP). In addition, the LP combination treatments showed excellent antibacterial activities *in vitro* and had protective effects against KP infections *in vivo*. Thus, this targeted combination provided a promising therapy strategy against KP infections such as improving spectrum of antibiotics or the treatment outcomes.

The membrane-disrupting activity of the polymyxin B, which enables the entry of a second antibacterial agent and subsequently increasing both permeability and antibacterial activity, is the critical synergism for the antibiotic combinations (Olsson et al., 2020). The absence of cross-resistance between linezolid and other protein synthesis inhibitors such as clindamycin against many MDR strains makes linezolid an attractive candidate to apply for combination treatments (Wasserman et al., 2016). To the best of our current knowledge, few studies have demonstrated the synergistic and antibacterial activities of LP against the KP *in vitro* and *in vivo*. A recent study has investigated the *in vitro* efficacy of linezolid in combination with polymyxin B against clinical CRKP (Wistrand-Yuen et al., 2020), while the potential toxicity of polymyxin B *in vivo* was probably remained. To reduce the toxicity of polymyxin B and retain its membrane-permeabilizing activity, we obtained three different derivatives of polymyxin B (PBHP, PBOP, PBNP). Additionally, we investigated the susceptibility of conventional anti-Gram-positive agents and different derivatives of polymyxin B against type strain KP or clinical CRKP in order to explore the synergistic activities of promising antibiotic combinations. Our results showed that the KP strains were resistant to the traditional anti-Gram-positive agents and polymyxin B derivatives, respectively. Nevertheless, the tested KP isolates were highly susceptible to linezolid combination of PBNP compared to other groups by checkerboard assay, suggesting that this drug combination was a potent candidate for treating KP infections. Since the combinations of LP had the lowest FIC index in all the tested group, we further investigated their antibacterial activity in the whole study.

In the current study, a significant difference was detected between monotherapy treatment and LP combination treatment against type strain KP or clinical CRKP by the time-kill assay. Although the bacteriostatic activity of PBNP was not sustained, linezolid in combination with PBNP showed the excellent antibacterial activity. These results were similar with several previous reports, in which the MDR-KP isolates were treated with polymyxin-based therapeutic combination (Pagès et al., 2015; Wistrand-Yuen et al., 2020). Although linezolid is a potent antimicrobial compound against lots of gram-positive pathogens, a recent follow-up study suggested that linezolid also exhibited the antibacterial activities against Gram-negative bacteria (Guzel Kaya et al., 2020). KP predominantly produce biofilms and can encapsulate bacteria layer by layer assembly and increase the bacterial tolerance to the current antibacterial agents (Tan et al., 2020). Interestingly, the LP combination treatment not only enhanced antibacterial activity, but also altered the biofilm production and morphology of KP, which might be effectively to prevent the colonization or spread of KP. Although the KP strains were highly insusceptible with PBNP alone, the morphology of KP was slightly altered after the PBNP alone treatment, suggesting that the bacteriostatic activity of PBNP was significantly reduced and the membrane-permeabilizing activity of PBNP was sustained. Altogether, these findings indicate that PBNP is the hopeful candidate of antibiotic potentiator and LP is probably excellent combination of antibacterial agents against KP.

To further investigate the protective effects of the LP against *K. pneumoniae* infection *in vivo*, the *C. elegans* were infected by KP or clinical CRKP and then treated with linezolid, PBNP alone or LP. Our results showed that the effective antibacterial activity against KP *in vivo* was detected by the LP combination treatment, since the linezolid and PBNP worked synergistically and the protective effects were improved as early as the second day of treatment. A very large number of bacteria were detected from the *C. elegans* in the monotherapy group or the control group, demonstrating that linezolid or PBNP alone could not provide the antibacterial activity *in vivo*. By contrast, the *C. elegans* treated with LP showed few bacteria, suggesting that most of bacteria were eliminated and the survival rate of the host was improved. Interestingly, treatment with LP also significantly decreased the CRKP burdens compared the control group and protected *C. elegans* against CRKP infection. Altogether, these data further confirmed the antibacterial activity of LP as we observed *in vitro* and indicated that linezolid could be the good alternative for the treatment of KP infections by combined with PBNP. Previous study showed that linezolid has activity against Gram-negative anaerobic bacteria (Yagi and Zurenko, 2003), while this study demonstrated that linezolid also has antibacterial activity against Gram-negative aerobic bacteria, which reinforced the interest in linezolid combined with polymyxin B derivatives against the other MDR Gram-negative bacteria. Thus, our data here provide evidential support that the PBNP could be an excellent potentiator in conjunction with linezolid to improve antibacterial activity and to protect the host against KP infections.

In conclusion, the combination of linezolid and PBNP showed significantly synergistic and antibacterial activities against the KP infection *in vitro* and *in vivo*. The presence of PBNP enhanced the antibacterial effects of linezolid, suggesting that PBNP could widen the therapeutic range of linezolid. Moreover, combination of the potent antibacterial agents in novel synergic formulations will provide a promising approach to inhibit the increase of bacterial resistance to the current antibiotics. Nevertheless, further studies to understand the impact of PBNP resistance are still in demand for detecting the ability of PBNP to work synergistically in combination with other available antibiotics. More importantly, we acknowledge that the low number of clinical strains examined or lack of dose-dependent investigation in LP combination treatment are the constraints of this study. Therefore, further more comprehensive or systematic study in rodents by evaluating the clinical practice of these findings may contribute to prevent the spread of *K. pneumoniae* infection in the long run.

## Data Availability

The original contributions presented in the study are included in the article/Supplementary Material, further inquiries can be directed to the corresponding authors.

## References

[B1] Bachman M. A. Breen P. Deornellas V. Mu Q. Zhao L. Wu W. (2015). Genome-Wide Identification of *Klebsiella pneumoniae* Fitness Genes during Lung Infection. mBio 6 (3), e00775. 10.1128/mBio.00775-15 26060277 PMC4462621

[B2] Chapelle C. Gaborit B. Dumont R. Dinh A. Vallée M. (2021). Treatment of UTIs Due to *Klebsiella pneumoniae* Carbapenemase-Producers: How to Use New Antibiotic Drugs? A Narrative Review. Antibiotics 10 (11), 1332. 10.3390/antibiotics10111332 34827272 PMC8615227

[B3] CLSI (2014). *Performance Standards for Antimicrobial Susceptibility Testing : Twenty-Fourth Informational Supplement.* CLSI Document M100-S24. Wayne, PA: Clinical and Laboratory Standards Institute.

[B4] de la Fuente-Núñez C. Reffuveille F. Mansour S. C. Reckseidler-Zenteno S. L. Hernández D. Brackman G. (2015). D-enantiomeric Peptides that Eradicate Wild-type and Multidrug-Resistant Biofilms and Protect against Lethal *Pseudomonas aeruginosa* Infections. Chem. Biol. 22 (2), 196–205. 10.1016/j.chembiol.2015.01.002 25699603 PMC4362967

[B5] Di X. Wang R. Liu B. Zhang X. Ni W. Wang J. (2015). *In Vitro* activity of Fosfomycin in Combination with Colistin against Clinical Isolates of Carbapenem-Resistant Pseudomas Aeruginosa. J. Antibiot. (Tokyo) 68 (9), 551–555. 10.1038/ja.2015.27 25805069

[B6] Doi Y. van Duin D. (2020). Polymyxin Resistance in *Klebsiella pneumoniae*: Complexity at Every Level. Clin. Infect. Dis. 70 (10), 2092–2094. 10.1093/cid/ciz627 31513703 PMC7201414

[B7] Guzel Kaya G. Medaglia S. Candela-Noguera V. Tormo-Mas M. Á. Marcos M. D. Aznar E. (2020). Antibacterial Activity of Linezolid against Gram-Negative Bacteria: Utilization of ε-Poly-l-Lysine Capped Silica Xerogel as an Activating Carrier. Pharmaceutics 12 (11), 1126. 10.3390/pharmaceutics12111126 PMC7700326 33233423

[B8] Huang T. Cui K. Song X. Jing J. Lin J. Wang X. (2019). MTOR Involved in Bacterial Elimination against *Trueperella Pyogenes* Infection Based on Mice Model by Transcriptome and Biochemical Analysis. Vet. Microbiol. 235, 199–208. 10.1016/j.vetmic.2019.06.021 31383303

[B9] Huang T. Pu Q. Zhou C. Lin P. Gao P. Zhang X. (2020). MicroRNA-302/367 Cluster Impacts Host Antimicrobial Defense via Regulation of Mitophagic Response against *Pseudomonas aeruginosa* Infection. Front. Immunol. 11, 569173. 10.3389/fimmu.2020.569173 33117356 PMC7576609

[B10] Kamaladevi A. Balamurugan K. (2017). Global Proteomics Revealed *Klebsiella pneumoniae* Induced Autophagy and Oxidative Stress in Caenorhabditis elegans by Inhibiting PI3K/AKT/mTOR Pathway during Infection. Front. Cel. Infect. Microbiol. 7, 393. 10.3389/fcimb.2017.00393 PMC5592217 28932706

[B11] Lawlor M. S. Hsu J. Rick P. D. Miller V. L. (2005). Identification of *Klebsiella pneumoniae* Virulence Determinants Using an Intranasal Infection Model. Mol. Microbiol. 58 (4), 1054–1073. 10.1111/j.1365-2958.2005.04918.x 16262790

[B12] Leach K. L. Swaney S. M. Colca J. R. McDonald W. G. Blinn J. R. Thomasco L. M. (2007). The Site of Action of Oxazolidinone Antibiotics in Living Bacteria and in Human Mitochondria. Mol. Cel. 26 (3), 393–402. 10.1016/j.molcel.2007.04.005 17499045

[B13] Li J. Chen X. Lin J. Yuan Y. Huang T. Du L. (2021). Antibiotic Intervention Redisposes Bacterial Interspecific Interacting Dynamics in Competitive Environments. Environ. Microbiol. 23 (12), 7432–7444. 10.1111/1462-2920.15461 33723911

[B14] Li J. Nation R. L. Turnidge J. D. Milne R. W. Coulthard K. Rayner C. R. (2006). Colistin: the Re-emerging Antibiotic for Multidrug-Resistant Gram-Negative Bacterial Infections. Lancet Infect. Dis. 6 (9), 589–601. 10.1016/s1473-3099(06)70580-1 16931410

[B15] Li R. Fang L. Tan S. Yu M. Li X. He S. (2016a). Type I CRISPR-Cas Targets Endogenous Genes and Regulates Virulence to Evade Mammalian Host Immunity. Cell Res 26 (12), 1273–1287. 10.1038/cr.2016.135 27857054 PMC5143421

[B16] Li X. He S. Li R. Zhou X. Zhang S. Yu M. (2016b). *Pseudomonas aeruginosa* Infection Augments Inflammation through miR-301b Repression of C-Myb-Mediated Immune Activation and Infiltration. Nat. Microbiol. 1 (10), 16132. 10.1038/nmicrobiol.2016.132 27670114 PMC5061341

[B17] Li X. He S. Zhou X. Ye Y. Tan S. Zhang S. (2016c). Lyn Delivers Bacteria to Lysosomes for Eradication through TLR2-Initiated Autophagy Related Phagocytosis. Plos Pathog. 12 (1), e1005363. 10.1371/journal.ppat.1005363 26735693 PMC4703367

[B18] Liu B. Liu Y. Di X. Zhang X. Wang R. Bai Y. (2014). Colistin and Anti-gram-positive Bacterial Agents against *Acinetobacter Baumannii* . Rev. Soc. Bras. Med. Trop. 47 (4), 451–456. 10.1590/0037-8682-0081-2014 25229285

[B19] Magi G. Marini E. Facinelli B. (2015). Antimicrobial Activity of Essential Oils and Carvacrol, and Synergy of Carvacrol and Erythromycin, against Clinical, Erythromycin-Resistant Group A Streptococci. Front. Microbiol. 6, 165. 10.3389/fmicb.2015.00165 25784902 PMC4347498

[B20] Olsson A. Wistrand-Yuen P. Nielsen E. I. Friberg L. E. Sandegren L. Lagerbäck P. (2020). Efficacy of Antibiotic Combinations against Multidrug-Resistant *Pseudomonas aeruginosa* in Automated Time-Lapse Microscopy and Static Time-Kill Experiments. Antimicrob. Agents Chemother. 64 (6), e02111. 10.1128/aac.02111-19 32179531 PMC7269485

[B21] Pagès J. M. Peslier S. Keating T. A. Lavigne J. P. Nichols W. W. (2015). Role of the Outer Membrane and Porins in Susceptibility of β-Lactamase-Producing Enterobacteriaceae to Ceftazidime-Avibactam. Antimicrob. Agents Chemother. 60 (3), 1349–1359. 10.1128/aac.01585-15 26666933 PMC4775948

[B22] Rajeshwari H. Nagveni S. Oli A. Parashar D. Chandrakanth K. R. (2009). Morphological Changes of *Klebsiella pneumoniae* in Response to Cefotaxime: a Scanning Electron Microscope Study. World J. Microbiol. Biotechnol. 25 (12), 2263–2266. 10.1007/s11274-009-0126-z

[B23] Stalker D. J. Jungbluth G. L. (2003). Clinical Pharmacokinetics of Linezolid, a Novel Oxazolidinone Antibacterial. Clin. Pharmacokinet. 42 (13), 1129–1140. 10.2165/00003088-200342130-00004 14531724

[B24] Tan S. Gao J. Li Q. Guo T. Dong X. Bai X. (2020). Synergistic Effect of Chlorogenic Acid and Levofloxacin against *Klebsiella Pneumonia* Infection *In Vitro* and *In Vivo* . Sci. Rep. 10 (1), 20013. 10.1038/s41598-020-76895-5 33203903 PMC7672055

[B25] Velkov T. Roberts K. D. Nation R. L. Thompson P. E. Li J. (2013). Pharmacology of Polymyxins: New Insights into an 'old' Class of Antibiotics. Future Microbiol. 8 (6), 711–724. 10.2217/fmb.13.39 23701329 PMC3852176

[B26] Wasserman S. Meintjes G. Maartens G. (2016). Linezolid in the Treatment of Drug-Resistant Tuberculosis: the challenge of its Narrow Therapeutic index. Expert Rev. Anti Infect. Ther. 14 (10), 901–915. 10.1080/14787210.2016.1225498 27532292

[B27] Wistrand-Yuen P. Olsson A. Skarp K. P. Friberg L. E. Nielsen E. I. Lagerbäck P. (2020). Evaluation of Polymyxin B in Combination with 13 Other Antibiotics against Carbapenemase-Producing *Klebsiella pneumoniae* in Time-Lapse Microscopy and Time-Kill Experiments. Clin. Microbiol. Infect. 26 (9), 1214–1221. 10.1016/j.cmi.2020.03.007 32224200

[B28] Wu M. C. Chen Y. C. Lin T. L. Hsieh P. F. Wang J. T. (2012). Cellobiose-specific Phosphotransferase System of *Klebsiella pneumoniae* and its Importance in Biofilm Formation and Virulence. Infect. Immun. 80 (7), 2464–2472. 10.1128/iai.06247-11 22566508 PMC3416469

[B29] Yagi B. H. Zurenko G. E. (2003). An *In Vitro* Time-Kill Assessment of Linezolid and Anaerobic Bacteria. Anaerobe 9 (1), 1–3. 10.1016/s1075-9964(03)00004-0 16887680

[B30] Ye Y. Tan S. Zhou X. Li X. Jundt M. C. Lichter N. (2015). Inhibition of P-Iκbα Ubiquitylation by Autophagy-Related Gene 7 to Regulate Inflammatory Responses to Bacterial Infection. J. Infect. Dis. 212 (11), 1816–1826. 10.1093/infdis/jiv301 26022442 PMC4633763

[B31] Zhao K. Li W. Li J. Ma T. Wang K. Yuan Y. (2019). TesG Is a Type I Secretion Effector of *Pseudomonas aeruginosa* that Suppresses the Host Immune Response during Chronic Infection. Nat. Microbiol. 4 (3), 459–469. 10.1038/s41564-018-0322-4 30617346

[B32] Zhou Y. F. Xiong Y. Q. Tao M. T. Li L. Bu M. X. Sun J. (2018). Increased Activity of Linezolid in Combination with Rifampicin in a Murine Pneumonia Model Due to MRSA. J. Antimicrob. Chemother. 73 (7), 1899–1907. 10.1093/jac/dky129 29897466

[B33] Zhuang Z. Wan D. Ding J. He S. Zhang Q. Wang X. (2020). Synergistic Activity of Nitroimidazole-Oxazolidinone Conjugates against Anaerobic Bacteria. Molecules 25 (10). 2431. 10.3390/molecules25102431 PMC7288012 32456032

